# Ribosomal Biogenesis and Heterogeneity in Development, Disease, and Aging

**DOI:** 10.3390/epigenomes7030017

**Published:** 2023-08-11

**Authors:** Rowshan Ara Islam, Charalampos Rallis

**Affiliations:** 1 School of Life Sciences, University of Essex, Wivenhoe Park, Colchester CO4 3SQ, UK; ri22394@essex.ac.uk; 2 School of Biological and Behavioural Sciences, Queen Mary University of London, Mile End Road, London E1 4NS, UK

**Keywords:** protein translation, ribosomes, ribosome biogenesis, stress, disease, growth, aging

## Abstract

Although reported in the literature, ribosome heterogeneity is a phenomenon whose extent and implications in cell and organismal biology is not fully appreciated. This has been the case due to the lack of the appropriate techniques and approaches. Heterogeneity can arise from alternative use and differential content of protein and RNA constituents, as well as from post-transcriptional and post-translational modifications. In the few examples we have, it is apparent that ribosomal heterogeneity offers an additional level and potential for gene expression regulation and might be a way towards tuning metabolism, stress, and growth programs to external and internal stimuli and needs. Here, we introduce ribosome biogenesis and discuss ribosomal heterogeneity in various reported occasions. We conclude that a systematic approach in multiple organisms will be needed to delineate this biological phenomenon and its contributions to growth, aging, and disease. Finally, we discuss ribosome mutations and their roles in disease.

## 1. Introduction

Ribosomes were discovered by George Palade and Albert Claude in the 1950s during their work on the structural and functional organisation of the cell, earning them the first Nobel Prize awarded in the field of physiology and medicine together with Christian de Duve in 1974 [1]. After 35 years, in 2009, studies of structure and function of ribosome garnered another Nobel Prize to V. Ramakrishnan, Thomas A. Steitz, and Ada E. Yonath [2]. At first, the newly discovered protein synthesis machinery was called the microsome; later, in 1958, it was renamed ‘ribosome’ at a symposium of the Biophysical Society [3]. Ribosomes have evolved in nature for billions of years. While maintaining a highly conserved core with increasing complexity from simple to complex organisms, ribosomal RNAs (rRNAs) hold the record of evolution in all domains of life [4]. In fact, the 18S rRNA of eukaryotic small subunit (SSU) is considered the basis of fundamental phylogenetic trees [5].

**Figure 1:**
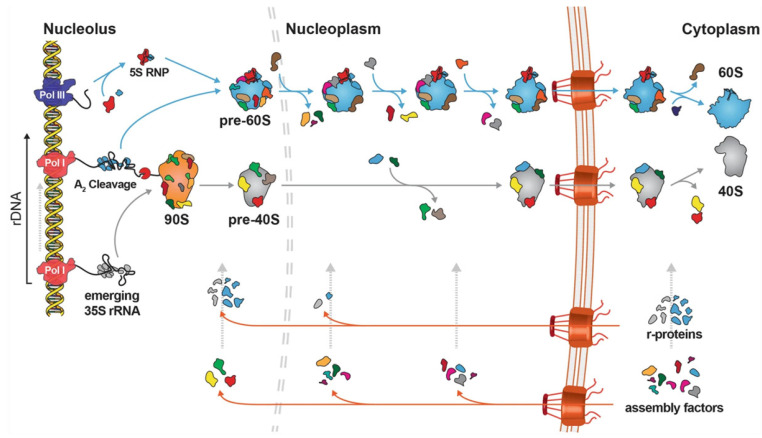
Ribosome biogenesis model in yeast. Transcription of primary 35S rRNA transcript, the common precursor of 18S, 5.8S, and 25S rRNAs, by Pol I from rDNA repeats, together with co-transcriptional joining of U3 snoRNP, r-proteins, and 40S assembly factors, form the 90S ribosomal precursor. 5S rRNA is transcribed separately by Pol III for assembly of 5S RNP before joining the 60S pre-ribosome. Co-transcriptional cleavage of 35S rRNA at the A2 site separates the 60S and 40S r-subunit maturation pathways. Pre-ribosomal particles undergo a cascade of maturation steps in the nucleoplasm by transient association with assembly factors until reaching nuclear export competency. Once exported to the cytoplasm, the last maturation events and quality control steps can occur, resulting in functional r-subunit production. (Adapted from [8] and licensed under the terms of the Creative Commons Attribution 4.0 International License, which permits use, sharing, adaptation, distribution, and reproduction in any medium or format. We thank Vikram Govind Panse for direct permission to use the figure. No alterations have been introduced from the source.).

Ribosome biogenesis is a spatially and temporarily dynamic process across the nucleolus, nucleoplasm, and cytoplasm. Multiple rDNAs stacked together code for precursor rRNAs that vary in the Svedberg unit of precipitation among species. In budding yeast, there are tandem repeats of rDNA containing 35S and 5S rDNA separated by intergenic spacer regions, where 35S pre-rRNA is the precursor for 5.8S, 18S, and 25S rRNAs [6,7]. In mammals, the analogous precursor is the 45S rRNA instead of 35S. Clusters of 60–800 copies of 45S precursor rDNA genes encoding 5.8S, 18S, and 28S rRNAs are located in the acrocentric chromosomes (chromosomes 13, 14, 15, 21, and 22 in humans) in nucleolus organiser regions (NORs). There are 10–400 copies of 5S rRNA located on chromosome 1; 45S/35S rDNAs are transcribed by RNA Pol I in the nucleolus, and 5S rDNA is transcribed by RNA Pol III generally in the nucleoplasm, with an exception in yeast, where it is likely to work in the nucleolus due to 5S rDNA’s interspersed location with 35S rDNA on chromosome XII [8,9,10]. RNA Pol II transcribes all ribosomal-protein-coding genes in the nucleoplasm. The mRNAs of ribosomal proteins (RPs) translocate to the cytoplasm and get translated, and, in most cases, RPs return to the nucleus. The precursor subunits are assembled in the nucleolus and in the nucleoplasm before being transported to the cytoplasm where they undergo further modifications to generate the mature and functional ribosomes [8,9]. Figure 1 illustrates the ribosome biogenesis process in yeast. While most RPs are assembled into the ribosome inside the nucleus, there are cases where a few specific RPs load onto the ribosome after they transport into the cytoplasm. For example, in the embryonic *Xenopus laevis* retinal ganglion cell, RPS4x/eS4, a RP important for axonal branching was observed to be translated and assembled on ribosomal surfaces locally in the axon, far from the nucleus. mRNAs of such RPs contain a short 5′ UTR, truncated at the CUIC motif/region, just upstream of the initiation codon, which is linked to nucleolus-independent assembly [11]. Ribosome biogenesis and protein synthesis are very ‘expensive’ cellular processes in terms of energy consumption and are not suitable, at least in high or normal levels, during cell division, stresses, or nutrient deficiency. Cells respond to oxidative stress, DNA damage, or amino acid level very fast by modulating the ribosome biogenesis as well as global protein translation rates, while growth and stress programs, with the first dependent on ribosomal availability, are opposing [12].

Both prokaryotic and eukaryotic ribosomes have two functional subunits, small (SSU) and large (LSU). The 30S prokaryotic SSU is composed of a 16S rRNA and 21 proteins, whereas the 50S LSU has two rRNAs, 5S and 23S, along with 31 proteins. In eukaryotes, the 40S SSU contains the 18S rRNA and 33 proteins. The eukaryotic LSU is made up of three rRNAs, 5S, 5.8S, and 25S/28S, and 49 proteins [13]. The rRNAs function as the catalytic core of the ribosome carrying approximately 55% of the ribosome mass and 80% of the total mass of cellular RNA content. Compared to prokaryotic rRNAs, eukaryotic rRNAs are larger and bear additional nucleotide sequences known as expansion segments (ES), 27 in the case of SSU rRNA and 59 for the LSU rRNAs, obtained over the period of evolution. Expansion segments interact with specific eukaryotic ribosomal proteins, and in some cases, their tentacle-like exposed parts recruit ribosome-associated proteins [4,14,15]. To start translation, the 16S rRNA in the prokaryotic SSU recognises the Shine–Dalgarno sequence in the ribosome-binding site (RBS) of mRNAs, while it is the Kozak consensus sequence in most eukaryotic mRNAs that works as the translation initiation site [16]. Translation starts with an aminoacylated tRNA interacting with the translation start codon on mRNA at the peptidyl-site (P-site) at the interface of small and large subunits. The first amino acid (methionine) at the P-site forms a peptide bond with the next amino acid brought to the aminoacylation site (A-site) by tRNA. The newly formed nascent peptide moves to P-site, emptying the A-site for the next amino acid. The deacylated tRNA leaves the ribosome via the exit site (E-site) [17]. The whole process ensures precise (although mistakes do happen) codon-by-codon reading and translation of the message carried by the bound mRNA [18].

## 2. Evolution of the Idea of Ribosome Heterogeneity and Its Key Factors

Soon after its discovery, Palade reported subtle differences among ribosomes that could not be resolved at that time due to the lack of appropriate techniques, approaches, and technological capabilities. Ribosome heterogeneity was proposed by other scientists too. The discoverer of DNA structure, Francis Crick, went even further by proposing the hypothesis of ‘one gene–one ribosome–one protein’ [19]. However further studies by Crick and Brenner suggested that ribosomes were non-specialised, passive, indiscriminate translation machines and that translation was controlled by mRNAs only [20]. The notion of ribosomal homogeneity remained unaltered until the 1980s when the study of model organisms resurfaced the idea of ribosome heterogeneity. In 1981, Ramagopal and Ennis observed a different stoichiometry of common ribosomal proteins and a few exclusively different ribosomal protein components between the vegetative amoebae and differentiated spore stages of *Dictyostelium discoideum* (slime mold) using 2-D gel electrophoresis [21]. Immense advancements in technical methods such as DNA and protein sequencing, translational profiling, and cryo-electron microscopy enabling atomic-level resolution have attracted more research towards unveiling the exact workings of ribosomes and mechanisms of translation together with their multi-layered regulation. Our current understanding is that the ribosomes are not only the effectors of translation process decoding mRNAs; rather than being a fixed supramolecular structure, ribosomes can be heterogeneous and may have specialised features to cater special needs within cells.

Ribosome heterogeneity may arise due to various factors, such as variation in rRNA sequences and their post-transcriptional modification, variable stoichiometry of RPs and their paralogues in different tissues and cellular development stages, and post-translational modification of RPs. In recent years, observation of ribosome heterogeneity in physiological and pathological conditions corroborated the idea of the specialised function of heterogeneous ribosomes.

In 2002, Mauro and Edelman found that before translation starts, the small subunit scrutinises and decides which mRNAs to translate and to what extent. This filtering preference may change with different heterogeneous ribosomes [22]. Generally, the 7-methylguanosine cap on the 5′ end of mRNAs interacts with the initiation factors to load onto the ribosome to start the translation process. However, recent studies showed that in special situations, such as stress when the initiation factors are repressed, the expansion segments (ES) of ribosomes may recognise mRNAs with 5′ internal ribosome entry sites (IRESs), shown in case of Hoxa9 mRNA by Maria Barna’s lab as a part of their detailed work on specialised ribosome [23]. First identified in viruses, IRES elements help translate viral mRNAs by recruiting the host’s cellular machinery in a cap-independent manner [24]. IRES-mediated translation was observed for selected cellular mRNAs when cap-dependent translation was downregulated (c-myc, XIAP, Apaf-1, p53 mRNAs) during stress [25], or sometimes this is a chosen means for some mRNAs (i.e., Hox mRNA with a translation inhibitor element (TIE) at 5′ UTR, which inhibits its cap-dependent translation in physiological condition) [25]. In the case of IRES-dependent translation of viral mRNAs, studies found RPS25 and RACK1 (ribosomal protein receptor for activated C kinase 1, an SSU protein) to be important in ribosomal composition [26,27]. But in the case of Hox mRNAs (transcribed from *Hox* genes responsible for embryonic body plan), ribosomes require RPL38, an otherwise dispensable ribosomal protein [25]. However, it is worth mentioning that a few recent studies contradicted with the idea of IRES-mediated cellular translation. One such study by McManus lab showed that most of the hyperconserved transcript leaders (hTLs), where the putative IRESes are, overlap transcriptional promoters, enhancers, and 3’splice sites, work as transcription factor binding site (E-box) for numerous transcription factors including USF1 and USF2, and the putative IRES sequences are rarely included in the transcript leader which argued the reported interaction of Hoxa9 IRES with ES (ES9S specifically) [28]. Their research attributed the putative IRES-like elements to mis-annotation and false positive result caused by monocistronic transcripts from internal promoters or cryptic splicing in the IRES test sequence in the bicistronic reporter assays [28]. As the McManus lab refuted the concept of IRES, the explanation of the observed IRES-like activity in this context can be found in a study done by Ivanov et al. [29]. Their study of ‘cap analysis of gene expression sequencing (CAGE-seq)’ of published data and mouse somites reported much shorter transcript leaders with conserved uORFs and absence of the putative IRESes in the Hox mRNAs. Translation may start at the start codon (AUG) or its near-cognate codon (CUG or UUG) at upstream ORF (uORF) or main ORF (mORF). Stringency of start codon selection depends on the flanking context nucleotides and the relative level of translation initiation factors eIF1 and eIF5. During high stringency (high level of eIF1 relative to eIF5, as seen during meiosis), Hox genes with ‘conserved poor mORF start codon context’ are inhibited, while mRNAs (Hoxa1, Hoxa9, Hoxa11) with ‘conserved inhibitory uORF with poor start codon context’ are induced. eIF1/eIF5 ratio is also increased during perturbation in global translation due to inhibition of ribosomal proteins (RPL11 in this study) which Ivanov et al. attributed as the reason of putative IRES containing Hox mRNAs’ connection with RPL38 reported by Barna group [29].

While heterogeneity of ribosomes is a natural means of translation regulation and may depend on cell type, growth, differentiation states, or cellular response to infections or other external stimuli [3,30,31], the association of certain RPs to special cellular conditions or specific mRNAs, such as the examples mentioned above, suggests the specialised roles of ribosomes. The concept of variable roles of individual RPs arises from the observation of different phenotypes caused by change in different proteins [5]. Comparative studies of phenotypes caused by loss-of-function mutations of RPs in eukaryotic organisms, namely, budding yeast, worm, drosophila, zebrafish, and mouse, showed a broad spectrum of phenotypes, including lethality, reduced organ/organism size, and delayed development. Haploinsufficiency due to the loss of one allele caused by mutation or deletion is more evident in tissues where the alleles of interest are more highly expressed [32]. RPs expressed selectively in certain cellular conditions and of varied stoichiometry are usually found on the surface of the ribosomes, near the mRNA entry/exit tunnel or L1 stalk, where they are in contact with the mRNAs [33].

Specific roles of individual RPs are also supported by the functional difference among RP paralogues. Even though paralogues are nearly identical in composition arising through gene duplication, their regulatory sequences and levels of expression vary, and important to the context of ribosome heterogeneity, their knockout causes different phenotypes [34]. Usually, one member of the paralogue pair is expressed dominantly in normal physiological conditions and is known as the major paralogue, in comparison to its minor counterpart, which is only expressed at special conditions. Paralogues may prefer different subsets of mRNAs, thus playing different roles. Usually, the major paralogue is hypoacetylated, reads shorter ORFs, and plays a house-keeping role, whereas the minor, hyperacetylated paralogue with a preference towards longer mRNAs contributes less to ribosome biogenesis and global cellular translation. For example, in a normal environment, the major paralogue of uL30 is a house-keeping ribosomal constituent in budding yeast. However, during an exposure to the drug staurosporine, the minor paralogue represses the major paralogue of uL30 and contributes towards upregulation and translation of long mRNAs of cell-wall-related proteins and thus contributes to drug resistance [5,34]. Post-translational modifications of RPs add an extra layer to ribosome heterogeneity. Usual modifications are methionine excision and acetylation at the N-terminus, as well as phosphorylation, acetylation, methylation, hydroxylation, and O-GlcNAc modification elsewhere within the molecule [5].

Like RPs, heterogeneity of rRNAs is also prevalent in all domains of life, across and within individuals, even in tissues. rRNA variation in eukaryotes is mostly in expansion segments (ES), along with some in the conserved region. A total of 35 different variations were discovered in the ES regions of 28S rRNA alone [22]. Variable rDNA copy number and their transcription level also add to heterogeneity [14]. Modification of rRNAs is important for efficient and accurate translation. Functionally important sequences are especially subjected to modifications and are located in areas within the vicinity of A, P, and E sites in order to ensure proper folding, optimum stability, and translation fidelity of the rRNAs. Apart from the general modification sites, there are tissue-specific modifications by snoRNAs. Co- and post-transcriptional modifications of rRNAs through snoRNAs could be the methylation of bases, methylation at 2′-hydroxyls, and uridine-to-pseudouridine conversion [35]. rRNA modification, namely, pseudouridylation, at certain sites, may impact IRES-dependent translation [36]. An example of specialised function of heterogeneous rRNA could be the loss of methylation at m5C2278 of 25S rRNA in yeast promoting polysomal recruitment of subsets of oxidative-stress-responsive mRNAs [37].

## 3. Ribosome Heterogeneity in Development

The rate of protein synthesis is generally significantly lower in stem cells compared to their differentiated counterparts. The rate of translation is high at the early stage of differentiation and gradually slows down at a later stage. This gradual change is regulated by lowering the synthesis of new rRNA and RP during the early stage for the low level of translation at later stages [14]. For normal development, the global or selective translation by ribosomes needs to be optimally synchronised with the cellular stages. Hypo-proliferation due to defective ribosomal proteins during development stages when high levels of global protein synthesis is required within a short period may cause delayed and anomalous development as seen in minute phenotypes of Drosophila. These include variable body size, thin bristles, and reduced fertility [38]. While hypo-proliferation due to reduction in global translation refers to the general role of ribosome in translation, effects caused by mutations in a few RPs call attention to their special roles. For example, mutation of uS5 causes death in *Drosophila* at the larval stages, while mutations of RPL5a (uL18) in zebrafish has been linked to defects in haematopoiesis and a malformed neurocranium. Mutations in the human orthologues for these two proteins cause Diamond–Blackfan anaemia 6 (DBA6), corroborating their similar roles across species [39].

Specialised roles of ribosomes, or the implication of specific ribosomal proteins in some processes, have been found, in several studies, to be critical in development, especially at the embryonic stage. The expression levels of certain RPs are used as markers for various cellular stages because of their signature expression pattern, which is also related to their specific roles apart from just being indiscrete constituents of the ribosome. For example, RPL14/eL14, RPL7A/eL8, RPL19/eL19, and RPL32/eL32 are considered as markers for inner cell mass of human blastocysts [40]. Upregulated RPL13 in the early blastomeres of humans and mice promotes translation and confers developmental competence to preimplantation embryos [41]. A recent study on human H7 embryonic stem cells (hESC) and on a mouse model reported a stricter selection and dynamic stoichiometry of 31 RPs at the surface and near the mRNA entry and exit points of polysomal ribosomes during the progression of differentiation. A total of 14 of these RPs showed at least 10% change relative to the undifferentiated hESC, which is a significant quantity considering the millions of ribosomes within a cell. These included RPL10A/uL1, RPL38/eL38, RPL11/uL5, RPS25/eS25, and RPS7/eS7 in mice and RPS25/eS25 and RPS10/eS10 in humans. Among these seven RPs with changed stoichiometry, RPL10A/uL1 was extensively studied [42]. This particular RP is associated with extracellular matrix organisation, system development, and steroid metabolism, and among its substrates with IRES elements are mRNAs coding for IGF2 (insulin-like growth factor 2), APP (amyloid beta precursor protein), and CHMP2A (charged multivesicular body protein 2A, involved in the endosomal sorting complex required for transport III-ESCRT III complex) [33]. During differentiation of hESC cells, RPL10A was upregulated in the primitive streak stage and gradually decreased as cells progressed to more differentiated mesodermal stages. Ribosomes containing RPL10A/uL1 translate the canonical and non-canonical Wnt pathway and other mesoderm regulators. Mice without this particular RP were characterised by the absence of the paraxial mesoderm and shortened posterior trunk [42]. Another LSU component, RPL38/eL38, appeared to be required for cap-independent translation of certain *Hox* mRNAs that encode proteins that are master regulators of vertebrate body plan, axis, and structure, as well as appendage morphogenesis, growth, and differentiation. Mutations in this RP have been associated with skeletal patterning defects and other abnormalities in mice. This phenomenon is comprehensible as RPL38 transcripts were found to be highly present in facial tissues; eyes; and the precursor cells of the brain, spinal cord, and axial skeleton [32]. Similar to RPL38, RPL24/eL24 may potentially be involved in skeletal (cranium, rib, cervical atlas) and eye formations, whereas the small subunit RPs uS12 and uS14 are linked to vasculogenesis, angiogenesis, and haematopoiesis [39]. As for the RP paralogues, studies found interesting data about their different expression levels, and in some cases, opposite roles in development. For example, in *Drosophila*, RpS5b, RpS10a, and RpS19b are enriched in germ-line cells in both males and females, suggesting their germ-line specific roles [38]. Rpl22 and Rpl22l1 paralogues in zebrafish manifest their opposing roles by binding SMAD1 mRNA but playing opposite effects on its translation with Rpl22 repressing and Rpl22l1 promoting during haematopoiesis in zebrafish embryos [43]. The same research group later reported an extraribosomal role of Rpl22 and Rpl22l1, where these proteins antagonistically controlled pre-mRNA splicing of SMAD2 during gastrulation [44].

Like RPs, heterogeneity of rRNAs during early embryogenesis has been reported. Some organisms utilise maternal rRNA sets during oogenesis and early embryogenesis, and then switch to the somatic rRNAs during later stages of embryogenesis. Differences in sequences and copy numbers (very high for maternal type) during these two developmental stages were observed for the 5S rRNA of *Xenopus* and zebrafish. During these two stages, the zebrafish 45S pre-rRNA (precursor of 5.8S 18S and 28S rRNA) originated from different chromosomal locations. The maternal-type SSU 18S rRNA, showed a preference for mRNAs expressed especially during oogenesis, whereas the somatic type 18S rRNA preferred mRNA expressed during the later stages of embryogenesis. In the case of 45S pre-rRNA, and unlike 5S rRNA, instead of a high copy number, additional DNA amplification was observed for the maternal type [45]. Similarly, the malarial parasite *Plasmodium berghei* expresses two different sets of rRNAs, the A-type rRNAs expressed during its development in rodents and the S-type within mosquitoes [46]. Whether the different sets of ribosomes contribute uniquely to the translatome of the developing embryo is a question from the perspective of the specialised ribosome. Heterogeneity caused by stoichiometric change in 2′-O-ribose methylation of rRNAs in *Dictyostelium discoideum* (slime mold) during different developmental stages and in response to environmental stimuli potentially contributed to different translatomes in various cellular conditions [47]. Changes in snoRNAs, and therefore their impact on rRNA modification, may affect the ribosomes: this is seen in zebrafish embryos, where a reduced level of snoRNAs (U26, U44, U78) resulted in a fatal reduction in methylation of their rRNA targets. Dysregulation of each of the snoRNAs resulted in different phenotypes. U44 morphants had a malformed trunk, U78 morphants showed a malformed hindbrain, and U26 morphants exhibited indistinct midbrain–hindbrain boundaries [35]. Aberration in Dyskerin, a pseudouridine synthase, affects IRES-dependent translation of certain mRNAs such as p27 and p53 in human and mouse cells [5].

Interestingly, no critical difference between rRNAs in the mouse oocyte and zygote stages was observed, even though the maternal ribosomes were replaced by the zygotic ones during maternal-to-embryo transition [48]. However, ribosome heterogeneity suggestive of specialised roles was observed in adult stages of mammals. For example, in the mouse brain, local protein synthesis in axons and dendritic spines of neurons are temporally controlled for growth and remodelling of synapses, enabling the brain’s development, recovery, plasticity, learning, and memory formation. In vitro and in vivo studies showed that in neurons, and particularly within axons and dendrites, ribosome composition was quite dynamic. Sleep, injury, or stress were proven to contribute to changes in ribosome composition. In activated hippocampal neurons (determined by the phosphorylation of RPS6/eS6), an experimental fear condition simulating a learning event after sleep resulted in elevation in 13 RP transcripts (RPS6, RPS12, RPS20, RPS21, RPS27, RPS28, RPS29, RPL21, RPL36A, RPL37, RPL37A, RPL39, RPL41) [49] (Table 1). In hippocampal excitatory neurons (CamKIIα+), seven RP transcripts (RPS9, RPS12, RPS19, RPS24, RPL19, RPL34, RPL35A) were reduced after an acute (5 h) period of sleep deprivation [50], while in dorsal root ganglion neurons 4 h following a sciatic nerve injury, 11 RPs (RPS12, RPS18, RPS19, RPS20, RPS25, RPL10A, RPL12, RPL14, RPL17, RPL27A, RPL32) were significantly reduced [51]. In terms of response to exogenous stimuli, exposure to oxidative stress with a 0.1 mM H_2_O_2_ treatment caused elevation of five RPs (RPS30, RACK1, RPL10, RPLP0, RPLP2) in primary culture [52].

## 4. Diseases Associated with Ribosomes and Relevant Mutations

In humans, ribosomopathies cover a spectrum of diseases caused by genetic defects in RPs, rRNAs, ribosome assembly factors, or RNA polymerases, resulting in disabling disorders as well as oncogenesis [55]. Differences in phenotypes due to different ribosomal mutations may point towards possible specialisations of ribosomes.

A large set of RPs are associated with a rare congenital disorder called Diamond–Blackfan anaemia, in which bone marrow failure causes a spectrum of pathologies including normochromic and macrocytic anaemia; reticulocytopenia; a near absence of erythroid progenitors in the bone; slow growth and reduced height; microcephaly and micrognathia (smaller head and lower jaw, respectively); and malformation of the upper limb, heart, and urinary systems [56]. Our review of the literature lists 29 RPs along with one ribosome-related protein called TSR2 ribosome maturation factor (TSR2) and three non-ribosomal proteins, namely, hematopoietic master transcription factor GATA1, erythropoietin (EPO), and adenosine deaminase 2 (ADA2), associated with this disease [57] (Table 2). Other ribosomopathies include refractory macrocytic anaemia (RPS14), autism/neurodevelopment disorder (RPL10), asplenia (RPSA), brachycephaly, trichomegaly and development delay (RPS23), spondyloepimetaphyseal dysplasia (poor bone growth) (RPL13), and hypotrichosis (poor hair growth) (RPL21) [39]. Examples of diseases indirectly affecting ribosome biogenesis are Shwachman–Diamond syndrome (*SBDS* gene affecting ribosomal maturation) and Treacher Collins syndrome (*TCOF1* gene affecting rRNA synthesis and pre-rRNA processing) [55]. 

Even though all ribosomopathies generally affect the bone marrow and therefore haematopoiesis, the severity of clinical presentation and treatment response vary, based on the affected ribosomal constituent [59]. Haploinsufficiency of RPs may cause faulty processing of pre-rRNAs, as RPs stabilise the important domains of rRNAs during their maturation. This theory is supported by the observation of lower levels of 45S precursor rRNA in DBA patients. Certain RPs may impact RNA polymerases, as observed the inhibition of RNA polymerase I during an experimental deficiency of RPS19 [60]. In general, all ribosomopathies share the common characteristics of hematopoietic dysfunction and/or skeletal malformation. The possible mechanism could be that changes in ribosomes negatively impact the transcriptome or specific sets of transcripts, required at the early stage of haematopoietic cells. This could be related or dependent on the GATA transcription factor, GATA1 [39]. Another mechanism could be inhibition of HDM2, an E3 ubiquitin ligase through imbalanced RPs and consequent dysregulated stabilisation of p53, resulting in apoptosis of erythrocytes [61]. Dysregulation of global translation in tissues with high demand or dysregulation of tissue-specific transcripts by altered ribosome components could also play a role.

Ribosomopathies pose 2.5–8.5-fold higher risk of developing cancer, reaching up to 200-fold of higher risk for certain cancers [55,62]. While DBA patients are at risk of cancers of the blood, bone, and colon, many ribosomopathies are prone to hematologic cancers only [63]. Prolonged mTORC1 activity, as seen in acute myeloid leukaemia, may be a possible cause of synthesis of oncogenic proteins in the backdrop of ribosomopathies [63]. In the context of specialised function of ribosomal constituents, in T-cell acute lymphoblastic leukaemia, loss of function of *RPL22* (and not its paralogue *RPL22L*, as mentioned later for glioblastoma) was reported in 10% cases, in association with the expression of stemness factor Lin28B via NF-kB, leading to cancer, referring to RPL22′s extraribosomal role [54]. In general, it seems paradoxical that compromised ribosome biogenesis in ribosomopathies is associated with tumorigenesis later, a phenomenon popularly known as the ‘Dameshek’s riddle’, proposed first by William Dameshek in 1967 [64]. Dameshek suggested that hypo-proliferative blood diseases caused by an insult to bone marrow were followed by repair processes, creating a subset of hyperproliferating cells. Considering the theory of molecular evolution, these repair processes could be reversion of the mutation, or mutations elsewhere, to bypass or overcome the effect of initial mutation. However, the latter case is not always beneficial as we know from recent research on the mechanisms of ‘Dameshek’s riddle’. For example, in Shwachman–Diamond syndrome, germline mutation of the *SBDS* gene, whose protein product removes EIF6 from the 60S subunit to help the association of 60S and 40S subunits to form the 80S subunit, causes the accumulation of free 60S subunits, leading to the activation of the TP53-mediated stress pathway [65]. In many cases the germline mutation of the *SBDS* gene is followed by an inactivating-somatic-mutation of EIF6 that bypasses the defective ribosome biogenesis. However, in a worse scenario, biallelic mutations in *TP53* were observed in SDS patients [66]. Even though the clonal selection of inactivating-*TP53*-mutation helps haematopoiesis, the absence of this important tumour suppressor may explain how diseases related to reduced ribosome biogenesis can lead to oncogenesis.

Interestingly, overexpressed RPs have also been reported in cancers, such as eL15/RPL15 in breast cancer, promoting cell cycle and metastasis [67], and in melanoma, reducing stress signalling and anti-tumorigenic response [68]. Low pH and lack of oxygen in tumour core promote the upregulation of specific RPs. These RPs may support carcinogenesis and progression or have an extra-ribosomal function, or even contribute to different isoforms of RPs that can support the translation of specific mRNAs, even when global translation is low. For example, the acidic condition of the tumour core in glioblastoma can reportedly lead to alternative splicing of *RPL22L1* to the *RPL22L1b* isoform that largely contributes to tolerance to acidosis, enhances stemness, and ultimately leads to poor prognosis [69]. uL29/RPL35 in neuroblastoma positively regulates the transcription factor HIF-1alpha to promote the Warburg effect [70]. As for the rRNAs, changes in snoRNAs can target many oncogenic transcription factors such as myc and cause aberrant modifications of rRNAs in cancer, resulting in enhanced yet faulty translation benefiting tumour growth [68].

Understandably, there are different hypotheses about ribosomopathies as well. One model suggested that mutated RPs result in ribosome shortage by causing an imbalance in the subunits’ ratio, leading to the reduced concentration of mature 80S ribosomes [71], while another implied that mutated RPs impact ribosome biogenesis via affecting rRNA processing, probably without being incorporated into the ribosome (in the case of DBA) [72]. In an experimental setting of imbalanced ribosomal subunits in yeast, Gregory et al. observed a higher rate of turn-over of excessive RPs that are not assembled in the ribosome and related proteins, instead of slowing down synthesis to adjust the ratio of subunits, causing wastage of cellular resources and possible implications in ribosomopathies [73]. Mills and Green suggested that during changed ribosome concentration, it is the mRNA-specific initiation rates that determine the cellular translational output [71].

## 5. Ageing and the Ribosome

The translation machinery in cells is downstream to the mTORC1 pathway. Inhibition of the mTOR pathway and the subsequent reduction in global protein translation are generally linked to an increase in lifespan [74,75,76,77,78,79]. A high level of translation simultaneously causes the depositon of toxic aggregates of damaged and misfolded proteins, resulting in increased cellular burden and stress. The reduction in global translation due to mTORC1 inhibition is often accompanied by selective translation of specifc proteins involved in maintenanace and damage repair. In aging cells, the overall protein synthesis decreases with an increased rate of incorporation of wrong amino acids into the newly synthesised proteins. The deletion of certain proteins (RPL3, RPL6b, RPL10, RPS6, and RPS18) of the ribosome in yeast showed an increase in lifespan, similar to that observed in mTORC1 inhibition in *wild*-*type* yeast cells. A comprehensive study in yeast by Nairita Maitra et al. with a particular focus on *RPL22* that has two paralogues, *RPL22a* and *RPL22b,* showed opposite roles of these paralogues in aging. Ribosomal composition was not affected by the loss of any of the *RPL22* paralogues, but mutants for *RPL22* paralogues have a different replicative lifespan (the number of mitotic divisions that a mother cell can undergo until it reaches quiescence and then senescence). *RPL22a∆* (deletion mutant) exhibited 38% longer life compared to *wild-type* and *RPL22b∆* mutants. Translational efficiency (ratio of the amount of the mRNAs that are bound to ribosomes to the total mRNA contained in cells) was found to be higher for GCN4 (a bZIP transcriptional activator of amino acid biosynthetic genes) in *RPL22a∆* compared to wild type and *RPL22b∆*. GCN4 contributed to longevity and increased the replicative lifespan of yeast. Even though translational efficiency was similar for mRNAs involved in cytoplasmic translation for both mutants, in *RPL22a∆* only, translational efficiency significantly decreased for serine and methionine metabolism enzymes of the one-carbon metabolism pathway, resulting in longer G1, slower growth, and sensitivity to oxidative stress [54]. Distinct ribosomal proteins, both of the LSU and the SSU, can contribute to an increase in yeast life span. Heterozygosity for *rpl10*, but not that of another LSU single copy RP gene, *rpl25*, led to a reduction of the translating ribosome population and increased replicative lifespan by 24% [80]. In the same study, the authors reported that ‘deletion of the SSU RPS6B paralog, but not of the RPS6A paralog increased replicative life span robustly by 45%, while deletion of both the SSU RPS18A, and RPS18B paralogs increased RLS moderately, but significantly by 15%’. These data indicated that specific ribosomal proteins might be able to modulate in a quantitative and qualitative manner the translation of proteins important in aging and possibly detoxification or even stress response [80].

While downregulation of many RPs was associated with increased lifespan, interestingly, overexpression of a few RPs did the same. The long-living *Drosophila* strains express a higher level of RPS28. When paralogues of RP28, RPS28a, and RPS28l were overexpressed in skeletal muscle cells, they translated a higher level of anti-aging mRNAs (mitochondrial function, stress resistance, DNA damage repair, autophagy) and showed a roughly 75% increase in lifespan [53]. Besides RPs, rRNAs, heterogeneous due to post-transcriptional modifications, play roles in aging. For example reduced cytosine methylation of 28S/25S rRNA in worms, flies, and yeast increased their lifespan [81].

Proteostasis (everything including protein synthesis, modification, quality control, and degradation) is pivotal during the life course and the aging process. Organisms lose their ability to maintain proteostasis and remove misfolded proteins systematically and chronically as they age. Promoter methylation of rRNAs and RPs, reduced levels of RNA polymerase I and translation factors, and the increase in global mRNA ‘noise’ have been reported to be evident in aged organisms. Ribo-seq analysis in mouse kidney and liver tissues across the life course shows a gradual decrease in dozens of components of ribosome biogenesis and the downregulation of the translation machinery at both the transcriptional and translational levels with age. In both tissues, an increase in inflammation and lysosomal proteins has been observed. Ribosome occupancy decreases with age in the liver for several SSU RPs (RPS5, RPS11, RPS21, RPS25), elongation factor EE2, and polyadenylate-binding protein 1 (PABPC1). The pattern of ribosome distribution on mRNAs also changes with aging [82].

Change in stoichiometry of ribosomal components with age has been reported as well. In young and aged yeast and mice, the composition of the lateral stalk of ribosome, a pentameric complex that includes the proteins RPLP0, RPLP1 an RPLP2, was found to be variable. In yeast, change in stalk composition rendered cells sensitive to cold, while in human cells, it was found to be linked to autophagy [5]. In mammalian senescent cells (senescent human HT1080 cells and aged mouse), the stalk region of polysomal ribosomes contained less RPLP2 proteins. The underlying mechanism was revealed to be a post-translational modification as the reduced levels of casein kinase 2 alpha (CK2α) or G-protein-coupled receptor kinase 2 (GRK2) rendered RPLP2 unphosphorylated and, therefore, unable to incorporate into the ribosome, causing detachment of the Y-box-binding protein-1 (YB-1), a critical transcription and translation regulator [83]. This finding was corroborated by a recent study in mice and humans, reporting a decreased level of YB-1 in bone marrow stromal cells with age, causing osteoporosis [84].

However, along with studies supporting the idea of ribosome heterogeneity and specialisation, there are findings nullifying it as well [85,86]. In a study using the short-lived vertebrate killifish (*Nothobranchius furzeri*), decoupling of transcriptome and proteome was evident in aging brains [87]. While transcriptome analysis showed increased mRNA level for all ribosomal proteins, protein levels increased for some, while they decreased for others. This result points to a possible loss of stoichiometry of ribosomal proteins (i.e., an imbalance in their relative levels) during aging, which is likely to impair ribosome assembly and to create a pool of orphan proteins at risk of aggregation. Using ribosome footprint data from young and old killifish brains, the authors showed that there are no changes in the translation output. These results pointed to other mechanisms of aging such as protein degradation and aggregation. In fact, age-related protein aggregates were enriched in ribosomal proteins, while proteasome activity in aging brains was reduced [87]. Other studies also claim that heterogeneity in ribosome stoichiometry and composition in aging is still debatable, especially in tissues such as the brain. In a study using quantitative proteomics and three different mouse brain tissues (hippocampus, cortex, and cerebellum), no significant changes across juvenile, adult, and middle-aged mouse groups were found. In all three brain tissues, an invariant set of 72 out of the 79 core RPs, RACK1, and two of the eight RP paralogs were detected. The data were compared with tissues of high metabolic rates (such as the liver), and the amounts were comparable there as well [88]. The examples chosen here showcase the absence of a clear picture for the contribution of ribosomal heterogeneity in the aging process itself. Future experimental design would be pivotal in addressing whether any changes in ribosome composition are causative effects of possible shorter lifespans or secondary effects of other fundamental processes. Nevertheless, even in the second scenario, it will be interesting to define the effects of ribosomal stoichiometry imbalances in bioenergetics, growth across the life course, and quantification of any possible changes in aging rates.

## 6. General Conclusions and Future Perspectives

Examples of ribosome heterogeneity are increasing in the literature, but the full appreciation and extent of the phenomenon is still elusive, together with a clear understanding in disease and aging. Recent advances in biomolecular interactions, genetic roadmaps, translatome studies, and structural approaches will be pivotal in understanding specialisation in various conditions such as in stress versus during growth, as well as young age versus old age. Of particular interest will be specialised ribosomes during development and in different tissues as well as in tumours, alongside possible differences between cancer stem cells that might have different cell-cycle patterns and metabolisms compared to fast amplifying cells of the cancerous population. Ribo-seq and Ribo-tag techniques will be pivotal in understanding the layers of gene expression regulation that are controlled through special ribosomes.

Important questions remain unanswered: what are the mechanisms by which different ribosomes affect translation? Do they affect global translation, or do they serve towards translating specific mRNAs? How does heterogeneity in ribosomes arise, and what are the cellular intrinsic and extrinsic factors that might regulate the formation of specialised ribosomes? Do specialised ribosomes persist in specific cell types or tissues? How dynamic and plastic are changes of ribosomal content when conditions (nutrients, stress, drug administration) change? A comprehensive and systematic approach in multiple experimental systems will be required to tackle this biological problem with the use of models from yeast to mouse and human cell lines or even patient-derived tissues or iPSC-derived cells.

## Figures and Tables

**Table 1:** Summary of reported roles of ribosomal subunits in development.

Biological Process	Organism	Associated Ribosomal Protein		Reference
		Cross Domain Name	Human Name	
Maintenance of germ-line cells	Drosophila	eS10	RPS10	[38]
		eS28	RPS28 (paralogues Rps28a and Rps28-like)	[53]
Maintenance of preimplantation embryo	mouse	eL13	RPL13	[41]
Mesoderm production	human embryonic stem cell	uL1	RPL10A	[42]
	mouse			
Eye development	mouse	eL24	RPL24	[39]
Neurocranium development	zebrafish	uL18	RPL5	[39]
Body plan/axial skeletal patterning	mouse	eL38	RPL38	[32]
Haematopoiesis and blood vessel formation	zebrafish	uS12	RPS23	[39]
		uS14	RPS29	
Haematopoiesis	zebrafish	eL22	RPL22 (paralogue RPL22l1)	[44]
		uL18	RPL5	[39]
Serine and methionine metabolism	yeast	eL22	RPL22 (paralogue RPL22a)	[54]
Circadian regulation	mouse	es12	RPS12	[50]
		eS19	RPS19	
		eS24	RPS24	
		uS4	RPS9	
		eL19	RPL19	
		eL33	RPL35A	
		eL34	RPL34	
Oxidative stress response	rat neuronal cells	eS30	RPS30	[52]
		RACK1	RACK1	
		P2	P2	
		uL10	P0	
		uL16	RPL10	
Learning and memory	mouse	es12	RPS12	[49]
		eS21	RPS21	
		eS27	RPS27	
		eS28	RPS28	
		eS6	RPS6	
		uS10	RPS20	
		uS14	RPS29	
		eL21	RPL21	
		eL36	RPL36	
		eL37	RPL37	
		eL39	RPL39	
		eL41	RPL41	
		eL43	RPL37A	

**Table 2:** Summary of ribosomal proteins and other proteins associated with diseases.

Disease	Associated Ribosomal Proteins		References
Diamond–Blackfan anemia	RP-SSU	RPS7, RPS8, RPS10, RPS15, RPS15A, RPS17, RPS19, RPS24, RPS26, RPS27, RPS27A, RPS28, RPS29	[56,57,58]
	RP-LSU	RPL3, RPL5, RPL7, RPL9, RPL11, RPL14, RPL15, RPL18, RPL19, RPL23A, RPL26, RPL27, RPL31, RPL35, RPL35A, RPL36	
	Other proteins	TSR2, GATA1, EPO, ADA2	[56]
Refractory macrocytic anaemia		RPS14	[39]
Autism, susceptibility to X-linked 5		RPL10	[39]
Isolated congenital asplenia		RPSA	[39]
Brachycephaly, trichomegaly, and development delay		RPS23	[39]
Spondyloepimetaphyseal dysplasia		RPL13	[39]
Hypotrichosis		RPL21	[39]
Shwachman–Diamond syndrome		Shwachman–Bodian–Diamond Syndrome (SBDS)	[55]
Treacher Collins syndrome		TCOF1	[55]

## Data Availability

Not applicable.
