# Correction: Islam, R.A.; Rallis, C. Ribosomal Biogenesis and Heterogeneity in Development, Disease, and Aging. *Epigenomes* 2023, *7*, 17

**DOI:** 10.3390/epigenomes7040026

**Published:** 2023-10-26

**Authors:** Rowshan Ara Islam, Charalampos Rallis

**Affiliations:** 1 School of Life Sciences, University of Essex, Wivenhoe Park, Colchester CO4 3SQ, UK; ri22394@essex.ac.uk; 2 School of Biological and Behavioural Sciences, Queen Mary University of London, Mile End Road, London E1 4NS, UK

## Citation Correction

23. Akirtava, C.; May, G.E.; McManus, C.J. False-Positive IRESes from Hoxa9 and Other Genes Resulting from Errors in Mam-malian 5’ UTR Annotations. *Proc. Natl. Acad. Sci. USA* **2022**, *119*, e2122170119. https://doi.org/10.1073/pnas.2122170119.

Should be revised to:

23. Leppek, K.; Fujii, K.; Quade, N.; Susanto, T.T.; Boehringer, D.; Lenarčič, T.; Xue, S.; Genuth, N.R.; Ban, N.; Barna, M. Gene- and Species-Specific Hox MRNA Translation by Ribosome Expansion Segments. *Mol. Cell* **2020**, *80*, 980–995.e13. https://doi.org/10.1016/j.molcel.2020.10.023.

In the original publication [1], the study by Akirtava et al was incorrectly cited instead of Leppek et al. Leppek et al and Ivanov et al were not cited in the original article. The correction reports the study by Leppek et al and Akirtava et al correctly and includes the reference from Ivanov et al to provide a different point of view about IRES reported by Leppek et al. The citation order of the references in the corrected manuscript have been adjusted accordingly following the numerical order after the change.

## Text Correction

As there are studies challenging the idea of an “Internal Ribosome Entry Site (IRES)” described in the original publication, it is ideal and fair to include the contradictory findings as well. Therefore, we added further text and relevant citations to Section 2.

**2. Evolution of the Idea of Ribosome Heterogeneity and Its Key Factors**

Paragraph Number 3. Additional text has been added and the paragraph should read:

In 2002, Mauro and Edelman found that before translation starts, the small subunit scrutinises and decides which mRNAs to translate and to what extent. This filtering preference may change with different heterogeneous ribosomes [22]. Generally, the 7-methylguanosine cap on the 5′ end of mRNAs interacts with the initiation factors to load onto the ribosome to start the translation process. However, recent studies showed that in special situations, such as stress when the initiation factors are repressed, the expansion segments (ES) of ribosomes may recognise mRNAs with 5′ internal ribosome entry sites (IRESs), shown in case of Hoxa9 mRNA by Maria Barna’s lab as a part of their detailed work on specialised ribosome [23]. First identified in viruses, IRES elements help translate viral mRNAs by recruiting the host’s cellular machinery in a cap-independent manner [24]. IRES-mediated translation was observed for selected cellular mRNAs when cap-dependent translation was downregulated (c-myc, XIAP, Apaf-1, p53 mRNAs) during stress [25], or sometimes this is a chosen means for some mRNAs (i.e., Hox mRNA with a translation inhibitor element (TIE) at 5′ UTR, which inhibits its cap-dependent translation in physiological condition) [25]. In the case of IRES-dependent translation of viral mRNAs, studies found RPS25 and RACK1 (ribosomal protein receptor for activated C kinase 1, an SSU protein) to be important in ribosomal composition [26,27]. But in the case of Hox mRNAs (transcribed from Hox genes responsible for embryonic body plan), ribosomes require RPL38, an otherwise dispensable ribosomal protein [25]. However, it is worth mentioning that a few recent studies contradicted with the idea of IRES-mediated cellular translation. One such study by McManus lab showed that most of the hyperconserved transcript leaders (hTLs), where the putative IRESes are, overlap transcriptional promoters, enhancers, and 3’splice sites, work as transcription factor binding site (E-box) for numerous transcription factors including USF1 and USF2, and the putative IRES sequences are rarely included in the transcript leader which argued the reported interaction of Hoxa9 IRES with ES (ES9S specifically) [28]. Their research attributed the putative IRES-like elements to mis-annotation and false positive result caused by monocistronic transcripts from internal promoters or cryptic splicing in the IRES test sequence in the bicistronic reporter assays [28]. As the McManus lab refuted the concept of IRES, the explanation of the observed IRES-like activity in this context can be found in a study done by Ivanov et al. [29]. Their study of ‘cap analysis of gene expression sequencing (CAGE-seq)’ of published data and mouse somites reported much shorter transcript leaders with conserved uORFs and absence of the putative IRESes in the Hox mRNAs. Translation may start at the start codon (AUG) or its near-cognate codon (CUG or UUG) at upstream ORF (uORF) or main ORF (mORF). Stringency of start codon selection depends on the flanking context nucleotides and the relative level of translation initiation factors eIF1 and eIF5. During high stringency (high level of eIF1 relative to eIF5, as seen during meiosis), Hox genes with ‘conserved poor mORF start codon context’ are inhibited, while mRNAs (Hoxa1, Hoxa9, Hoxa11) with ‘conserved inhibitory uORF with poor start codon context’ are induced. eIF1/eIF5 ratio is also increased during perturbation in global translation due to inhibition of ribosomal proteins (RPL11 in this study) which Ivanov et al. attributed as the reason of putative IRES containing Hox mRNAs’ connection with RPL38 reported by Barna group [29].

To adjust with the new insertion in Section 2. Evolution of the Idea of Ribosome Heterogeneity and Its Key Factors, Paragraph Number 3, we will move the last sentence of Paragraph 3 of original publication to the beginning of Paragraph 4 of the original publication, and should read:

While heterogeneity of ribosomes is a natural means of translation regulation and may depend on cell type, growth, differentiation states, or cellular response to infections or other external stimuli [3,30,31], the association of certain RPs to special cellular conditions or specific mRNAs, such as the examples mentioned above, suggests the specialised roles of ribosomes. The concept of variable roles of individual RPs arises from the observation of different phenotypes caused by change in different proteins [5]. Comparative studies of phenotypes caused by loss-of-function mutations of RPs in eukaryotic organisms, namely, budding yeast, worm, drosophila, zebrafish, and mouse, showed a broad spectrum of phenotypes, including lethality, reduced organ/organism size, and delayed development. Haploinsufficiency due to the loss of one allele caused by mutation or deletion is more evident in tissues where the alleles of interest are more highly expressed [32]. RPs expressed selectively in certain cellular conditions and of varied stoichiometry are usually found on the surface of the ribosomes, near the mRNA entry/exit tunnel or L1 stalk, where they are in contact with the mRNAs [33].

The authors state that the scientific conclusions are unaffected. This correction was approved by the Academic Editor. The original publication has also been updated.
